# Neutrophils in Tissue Injury and Repair: Molecular Mechanisms and Therapeutic Targets

**DOI:** 10.1002/mco2.70184

**Published:** 2025-04-21

**Authors:** Luying Yang, Fan Shi, Feng Cao, Le Wang, Jianzhen She, Boling He, Xiaoying Xu, Liang Kong, Bolei Cai

**Affiliations:** ^1^ Department of Oral and Maxillofacial Surgery State Key Laboratory of Oral & Maxillofacial Reconstruction and Regeneration National Clinical Research Center for Oral Diseases Shaanxi Key Laboratory of Stomatology School of Stomatology The Fourth Military Medical University Xi'an China

**Keywords:** neutrophils, polarization, angiogenesis, tissue repair, neutrophils fate

## Abstract

Tissue repair represents a highly intricate and ordered dynamic process, critically reliant on the orchestration of immune cells. Among these, neutrophils, the most abundant leukocytes in the body, emerge as the initial immune responders at injury sites. Traditionally recognized for their antimicrobial functions in innate immunity, neutrophils now garner attention for their indispensable roles in tissue repair. This review delves into their novel functions during the early stages of tissue injury. We elucidate the mechanisms underlying neutrophil recruitment and activation following tissue damage and explore their contributions to vascular network formation. Furthermore, we investigate the pivotal role of neutrophils during the initial phase of repair across different tissue types. Of particular interest is the investigation into how the fate of neutrophils influences overall tissue healing outcomes. By shedding light on these emerging aspects of neutrophil function in tissue repair, this review aims to pave the way for novel strategies and approaches in future organ defect repair, regeneration studies, and advancements in tissue engineering. The insights provided here have the potential to significantly impact the field of tissue repair and regeneration.

## Graphical abstract

Tissue repair is a highly intricate and ordered dynamic process that heavily relies on the orchestration of immune cells. In addition to their well‐established antimicrobial functions in innate immunity, neutrophils also play an indispensable role in tissue repair. In this review, we focus on the emerging roles of neutrophils in the early stages of tissue injury, encompassing their recruitment and activation mechanisms following tissue damage, their involvement in vascular network formation, their contribution to tissue repair processes, and the impact of neutrophil fate on overall tissue healing outcomes

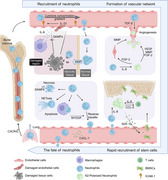

## 1 Introduction

Tissue repair is a complex ‐ dynamic process. After tissue damage, the body initiates a series of mechanisms for self‐repair, including the recruitment of immune cells, clearance of damaged cells, migration of repair‐related cells such as stem cells, proliferation and differentiation of cells in the injured area, deposition and remodeling of extracellular matrix (ECM), and so on [1]. The precise regulation of these stages largely depends on the role of immune cells. As the body's response is often transmitted downward through cascading amplification reactions, we believe that the influence of early immune cells on the outcome of repair is greater [2].

Neutrophils are a type of polymorphonuclear leukocyte. As the predominant immune cell population in the body, they constitute approximately 60–70% of all leukocytes in human blood and serve as the primary responders of the innate immune system. Structurally, they have a multilobed nucleus, typically consisting of 2–5 connected lobes, which allows them to be flexible and migrate through tissues. Neutrophils exhibit a double‐edged characteristic in various inflammatory diseases owing to their heterogeneity. Their cytoplasm contains granules filled with enzymes and antimicrobial proteins, such as lysozyme, defensins, and myeloperoxidase (MPO), that assist them in destroying pathogens [3]. Their bactericidal functions in combating invading pathogens have been well established, including phagocytosis, release of cytotoxic granules, generation of reactive oxygen species (ROS), and formation of neutrophil extracellular traps (NETs). However, in this process, the acute inflammation triggered by neutrophils can also lead to ECM degradation, tissue damage, and inhibition of tissue regeneration [4, 5]. Therefore, neutrophils have long been considered inflammatory cells that trigger acute inflammation. In recent years, with further research, a large body of evidence suggests that neutrophils' anti‐inflammatory function also play an important role in tissue repair and regeneration. As the cells are recruited earliest to the site of injury, their initial inflammatory response at the site of tissue damage significantly influences the outcome of tissue repair [6]. Our team recently discovered that neutrophils mediate the recruitment of stem cells and other immune cells in the early stages of bone injury/regeneration, subsequently triggering important processes in tissue repair such as proliferation of repair‐related cells, reconstruction of the vascular network, and remodeling of the ECM [6, 7, 8].

**FIGURE 1:**
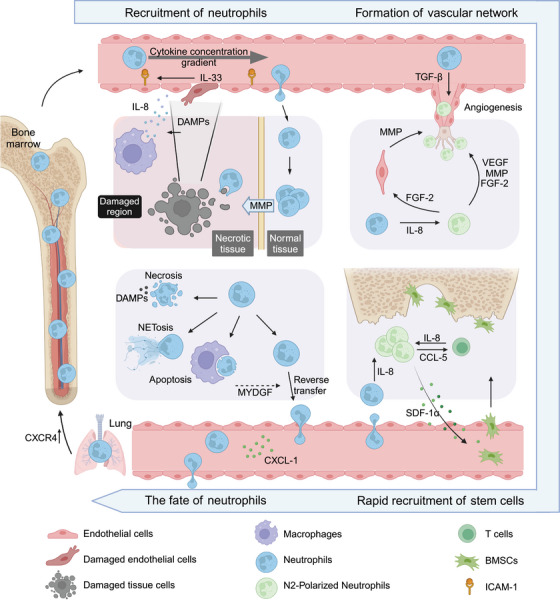
The role of neutrophils in tissue repair. Using bone tissue injury repair as an example: (1) After tissue injury, damaged cells release damage‐associated molecular patterns (DAMPs), which directly recruit neutrophils and induce local macrophages and other immune cells to release chemokines such as interleukin 8 (IL‐8) and damaged endothelial cells to release interleukin 33 (IL‐33). IL‐33 and IL‐8, along with other chemokines, create a gradient that guides the local infiltration of neutrophils; Neutrophils entering the injury site break through the barrier of the necrotic zone by using matrix metalloproteinases (MMPs) to clear cellular debris; (2) An appropriate concentration of IL‐8 can promote the recruitment of a large number of N2‐polarized neutrophils to the defect area. N2 neutrophils secrete angiogenic factors such as vascular endothelial growth factor (VEGF), matrix metalloproteinase 9 (MMP9), fibroblast growth factor 2 (FGF2), among others. FGF2 stimulates endothelial cells to produce MMPs, promoting the migration of endothelial cells and perivascular cells, rebuilding the damaged vascular network. (3) Additionally, N2 neutrophils can release stromal cell‐derived factor 1 (SDF‐1) to recruit bone marrow mesenchymal stem cells (BMSCs) to participate in tissue repair and regeneration; (4) Neutrophils, as short‐lived cells, exhibit post‐repair fates such as necrosis, NETosis, apoptosis, and reverse migration. The neutrophil fate has a significant impact on the outcome of tissue repair. Among them, neutrophils undergoing reverse migration re‐enter the vascular system, pass through the lungs, and return to the bone marrow, where they are cleared; this process is considered the most favorable outcome for tissue repair. This figure was created using BioRender.com.

In this review, we focus on the emerging role of neutrophils in tissue repair and summarize the key roles of neutrophils in the tissue repair process: how neutrophils are recruited and activated after tissue injury, how neutrophils promote the formation of the vascular network, how they participate in the tissue/organ repair, and how the fate of neutrophils affects the outcome of tissue repair (Figure 1). We systematically analyze the diverse molecular mechanisms underlying neutrophil‐mediated regeneration and extensively explored the potential of targeting neutrophils for inflammation resolution and tissue repair.

## 2 Recruitment of Neutrophils in Tissue Injury Area

### 2.1 Recruitment Process

After tissue injury, the damaged area releases a series of molecular signals ‐ including damage‐associated molecular patterns (DAMPs) initiated by damaged cells or pathogen‐associated molecular patterns (PAMPs) triggered by external invaders ‐ that induce rapid neutrophil recruitment to the injury site [9].

**FIGURE 2:**
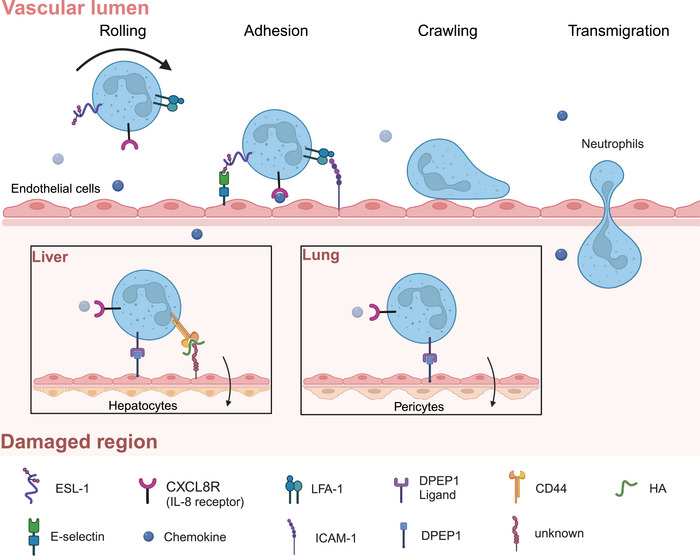
The process of neutrophil recruitment. First, neutrophils are released from the bone marrow, while endothelial cells upregulate selectins (E‐selectin) and integrin ligands (e.g., intercellular adhesion molecule 1 (ICAM‐1)). Subsequently, free neutrophils exhibit high expression of adhesion‐related proteins, including the E‐selectin receptor–ESL‐1, the chemokine receptor CXCL8R, and LFA‐1. These proteins facilitate the adherence and crawling of neutrophils on endothelial tissues via receptor‐ligand interactions. Ultimately, neutrophils transmigrate through the endothelial barrier to reach the damaged region. In cases where selectins or integrins are absent or mutated, neutrophil recruitment in organs such as the liver and lung can be mediated by Dipeptidase ‐ 1 (DPEP1). Specifically, in the inflamed liver, recruited neutrophils bypass the rolling phase and directly adhere to the injured area through the interaction between CD44 and hyaluronic acid (HA) on liver sinusoidal endothelial cells. This figure was created using BioRender.com.

The classical process of neutrophil recruitment, including rolling, adhesion, crawling, and transmigration, predominantly occurs in muscle, skin, and mesentery tissues [10]. This phenomenon is accomplished through the selectin‐integrin axis. Specifically, endothelial cells secrete selectins (e.g., E‐and P‐selectin) that interact with selectin receptors on neutrophils (e.g., E‐selectin ligand 1 [ESL‐1] and P‐selectin glycoprotein ligand 1‌‌ [PSGL‐1]), facilitating the initial tethering and rolling of neutrophils. Subsequently, modified neutrophils bind to a family of integrin molecules such as leukocyte function‐associated antigen ‐ 1 (LFA ‐ 1) and macrophage‐1 antigen, leading to their recruitment at the site of injury [10, 11]. However, in the inflamed liver, recruited neutrophils do not roll, but instead adhere directly to the damaged area through the interaction of CD44 and hyaluronic acid on the hepatic sinusoidal endothelial cells [12]. The accumulation of neutrophils in the lungs can still occur despite selectin/integrin deficiency or functional mutations, indicating that alternative mechanisms may be involved [13, 14]. Dipeptidase ‐ 1 (DPEP1) functions as an adhesion receptor and mediates the recruitment of neutrophils in the liver and lungs, independent of its dipeptidase activity [15]. As the initial influx of immune cells infiltrates the injury zone, neutrophils express a variety of receptors on their surface: G protein‐coupled receptors (GPCRs), Fc receptors, adhesion receptors, cytokine receptors, and pattern recognition receptors (PRRs), through which they recognize and respond to damage and infection (Figure 2) [16, 17].

The cascade reaction of neutrophil recruitment triggered by DAMPs is a finely regulated process in the body. DAMPs are recognized by neutrophils through PRRs and subsequently activate multiple signaling pathways, thereby inducing neutrophil activation. Toll‐like receptor 4 (TLR4) activation is typically mediated by myeloid differentiation factor 88 (MyD88), which in turn activates tumor necrosis factor (TNF) receptor‐associated factor 6 (TRAF6) and promotes the production of inflammatory factors such as TNF‐α, IL‐1β, and IL‐8 via the nuclear factor kappa‐B (NF‐κB) pathway [18]. IL‐8 serves as the primary chemokine for neutrophils and facilitates their migration to sites of injury [19]. Additionally, DAMPs can activate the NLRP3 inflammasome complex to induce Caspase‐1‐mediated maturation and secretion of IL‐1β and IL‐18 [20]; this further enhances neutrophil activation and amplifies their inflammatory response. Furthermore, the release of DAMPs by damaged cells elicits the activation of resident cells within the adjacent tissues, encompassing macrophages, dendritic cells, mast cells, and endothelial cells. This activation prompts the robust secretion of a plethora of chemokines, thereby orchestrating the precise guidance of neutrophil recruitment toward the site of injury. The chemokines include biologically active lipid metabolites, N‐formyl methionyl‐leucyl‐phenylalanine (the first leukocyte chemoattractant peptide discovered), complement components, and various cytokines [5, 21]. For instance, the serum metabolite 5‐HIAA, generated by activated platelets and mast cells, binds to the GPCR GPR35 and facilitates recruitment of neutrophils [22]. Once PAMPs are recognized by immune cells, they induce the activation of nuclear transcription factors through intracellular signaling pathways, leading to the secretion of cytokines such as IL‐1, IL‐6, and TNF‐α by macrophages and neutrophils. This triggers an acute inflammation characterized by intense neutrophil infiltration, which serves to defend against foreign invaders while causing tissue damage [23, 24]. Two important mechanisms involve the extensive release of NETs from the nucleus to restrict microbial spread, as well as the degranulation process that involves the release of MPO to kill or inhibit microbes [25]. (There is extensive research on this topic, so I will not go into further detail here.) The early influx of neutrophils into the injury site establishes a good foundation for the construction of the early microenvironment of tissue repair by clearing necrotic cells/tissues and promoting vascular network formation, which is a critical link in tissue injury repair [26].

### 2.2 Cytokines that Drive Recruitment of Neutrophils

Throughout the complete injury‐to‐tissue repair cycle, multiple phases occur, encompassing the inflammatory response phase, wound healing phase, and tissue remodeling phase. Following stimulation with potent chemoattractants such as interleukin‐8 (IL‐8; also known as C‐X‐C motif chemokine ligand 8 [CXCL8]) and IL‐33, neutrophils are recruited as the initial cell population at inflamed sites and exhibit chemotactic migration from peripheral blood circulation to tissues to fulfill their functional role.

#### 2.2.1 IL‐8 (CXCL8)

IL‐8, previously referred to as neutrophil chemotactic factor, is a well‐known cytokine with the highest known activity in neutrophil chemotaxis and can be released by various cell types [19, 27]. Following the release of DAMPs by cells in the injury site, local tissue cells and resident macrophages are induced to release IL‐8 and other potent neutrophil chemotactic factors. The released IL‐8 in the injury site can bind to glycosaminoglycans on cell walls and ECM, generating a chemotactic gradient. By binding to the two heterotrimeric GPCRs, CXCR1 and CXCR2, IL‐8 guides the local infiltration of neutrophils, with the chemotactic gradient formed by this vascular and ECM binding reaching up to 650 µm [21, 28]. Furthermore, our latest research has revealed that only an appropriate concentration of IL‐8 can induce ectopic ossification within mouse muscle. Inadequate IL‐8 concentrations fail to guide regeneration, while excessive levels of IL‐8 may lead to local tissue damage. The underlying mechanism for these outcomes may be directly associated with the polarization direction (activation level) of recruited neutrophils [8]. In addition, the function of IL‐8 in promoting tissue repair has been gradually discovered, such as accelerating the formation of new blood vessels by guiding the migration of endothelial cells and endothelial precursor cells and inducing the differentiation of stem cells into cartilage [29, 30].

#### 2.2.2 Interleukin‐33

IL‐33, a member of the IL‐1 family, is secreted by various cells such as endothelial cells, epithelial cells, fibroblasts, keratinocytes, and macrophages, playing a crucial role in the body's immune response [31]. During tissue damage, the barrier function and integrity of endothelial cells are disrupted, leading to the massive release of IL‐33 as an “alarm signal” from the damaged endothelial cells [31]. IL‐33 activates the ST2 receptor (suppression of tumorigenicity), promoting neutrophil adhesion and enhancing their recruitment [32]. Additionally, IL‐33 can activate mast cells and T cells beneath local blood vessels. Activated mast cells produce cytokines such as TNF, IL‐1, and IL‐6 to increase the expression of ICAM‐1 on endothelial cells, promoting neutrophil adhesion. They also release chemokines like CXCL1, CXCL2, and CXCL8 to enhance neutrophil recruitment [31, 33‐36]. IL‐33 recruits and activates T cells, which differentiate into Foxp3^+^CD4^+^ regulatory T cells under the guidance of neutrophils. These regulatory T cells modulate local immune responses while secreting IL‐8 to recruit neutrophils [37]. Activated neutrophils release chemokines like CCL‐5 to recruit T cells, which further secrete IL‐8 to recruit more neutrophils, forming a positive amplification loop to maintain neutrophil concentration in the injured area and provide a sufficient cellular basis for early tissue repair [38]. The cascade amplification reaction formed during tissue repair creates conditions for the spatiotemporal recruitment of various functional cells needed for tissue repair, suggesting that early regulation is a more efficient therapeutic strategy. However, tissue repair is complex, and how the cascade amplification reaction is regulated and inhibited in later stages is still unclear. Improper regulation of the cascade amplification reaction can lead to excessive activation of neutrophils, ultimately resulting in organ fibrosis [39].

## 3 Neutrophils Promote Vascular Network Reconstruction

The reconstruction of the vascular network in the injury site is a critical step in the tissue repair process. The neovascular network participates in the formation of granulation tissue, providing nutrients for the regeneration and repair of damaged tissues, and also provides a path for the migration and recruitment of circulating stem cells [40, 41]. Vascular network reconstruction is a dynamic process highly regulated by environmental signals [40]. Increasing evidence suggests that neutrophils can promote vascular network reconstruction and tissue repair by remodeling the ECM in the injury site and constructing a regenerative microenvironment.

### 3.1 Neutrophils Break Through the Barrier of the Injury Site by Matrix Metalloproteinases

Matrix metalloproteinases (MMPs) are a class of highly conserved enzymes composed of a series of proteinases that are dependent on metal ions such as zinc or calcium, hence the name [42]. Among them, MMP9 is known to play a crucial role in remodeling the ECM and promoting vascular formation. MMP9 not only degrades the ECM, transforming it into a low‐density primitive state that is more conducive to cell migration, proliferation, and angiogenesis, but it also releases vascular endothelial growth factor (VEGF) and other growth factors bound to the ECM, thereby promoting angiogenesis and tissue growth [43]. As neutrophils are among the first cells to arrive at the site of injury, they transport highly active MMP9 to the injured tissue, accelerating the remodeling of the local ECM and the re‐establishment of the vascular network [42]. In 2015, a study published in “Science” reported that neutrophils can break through the barrier surrounding the damaged liver tissue by secreting various MMPs during liver tissue injury. This process guides the growth of new blood vessels into the injured area, ultimately promoting the repair of the damaged liver [44]. Therefore, neutrophils in the early stages of tissue injury secrete large amounts of highly active MMP9 to break through the barrier between the injured area and normal tissue, while also guiding the growth of new blood vessels into the injured area, creating a pathway for repair‐related cells to enter the damaged area. MMP9 also remodels the ECM in the injured area, transforming it into a more loosely structured primitive state, thereby creating a favorable local microenvironment for building the vascular network and tissue regeneration in the damaged area.

### 3.2 Neutrophils Promote Vascular Network Construction by Activating VEGF

Neutrophils are one of the essential immune cells involved in promoting vascular network construction. Research has found that in mouse injury models, neutrophils that are among the first to enter the injured area, as well as local endothelial cells, highly express VEGF, which accelerates the formation of blood vessels [45]. VEGF, regarded as one of the paramount growth factors, is competent in stimulating glycolysis by elevating the expression levels of glucose transporter 1, fructose‐2,6‐bisphosphatase‐3, and lactate dehydrogenase‐A [46, 47, 48, 49, 50]. Additionally, the activation of VEGFR2 initiates signaling via the PI3K/Akt pathway [50]. When neutrophils are depleted, the local production of vascular growth factors is completely inhibited, and endothelial cells no longer express VEGF [51]. This indicates that neutrophils can directly secrete VEGF, a proangiogenic factor, and also induce endothelial cells to produce VEGF, leading to high local expression of VEGF, which in turn accelerates the construction of the vascular network and promotes injury repair.

### 3.3 The Subtype of Neutrophils that Promote Vascular Network Construction

In recent years, tumor pathology has defined two subtypes of neutrophils that exhibit phenotypic and functional differences: the N1 phenotype with antitumor properties and the N2 phenotype with protumor properties. Based on their ability to degranulate, release cytokines, and migrate, they are further classified into proinflammatory (N1) and anti‐inflammatory (N2) types [52, 53, 54, 55]. The transcriptomic analysis confirmed N1 and N2 neutrophils to be separate populations with a short life span, and the proinflammatory milieu triggers the polarization of N1 neutrophils while the anti‐inflammatory microenvironment promotes N2 polarization. The N1 phenotypes augment the proinflammatory response by secreting elevated levels of interferon (IFN)‐γ, IL‐1β, IL‐6, and TNF‐α, by generating ROS, and ultimately through NETosis [56, 57]. However, The exposure of neutrophils to transforming growth factor β (TGF‐β) or granulocyte colony‐stimulating factor (G‐CSF) was shown to induce the polarization toward the N2 phenotype that produce fibrotic or reparative proteins such as TGF‐β1 and arginase [53, 58‐61].  The precise mechanism through which they undergo transformation remains uncertain. Sensory neurons in the lungs modulate inflammation and the function of neutrophils through the release of neuropeptides, such as calcitonin gene‐related peptide (CGRP) [62]. Research has revealed that Mas‐related of GPCR a1 (Mrgpra1) are associated with neuropeptide FF (NPFF), which have the ability to direct neutrophil specialization in cases of bacterial pneumonia [63]. NPFF‐producing neutrophils suppress a proinflammatory state via activation of Mrgpra1, resulting in an Arginase‐1 (Arg1) anti‐inflammatory phenotype, whereas the loss of Mrgpra1 in neutrophils drives an IFNγ‐dependent proinflammatory phenotype [63]. It has also been verified that exosomes originated from human umbilical cord mesenchymal stem cells (HucMSC‐Exo) are capable of activating the Jak2/STAT6 pathway. This activation mediates the mitochondrial metabolic reprogramming within neutrophils and potentiates their transformation into N2 prorepair subtypes [64].The genetic structures of neutrophils undergo a reprogramming process in both acute and chronic inflammation, triggered by external signals (such as TGF‐β‐mediated), leading to dynamic transitions between subpopulations [65]. Namely, N2 neutrophils increase over time in tissue repair, and the decrease in the N1/N2 ratio is directly associated with the resolution of inflammation [59].

Among them, N2 neutrophils with immunoregulatory functions play a significant role in vascular remodeling. Although the specific mechanisms underlying neutrophil polarization are still being elucidated, research has found that N2 neutrophils secrete proangiogenic factors such as VEGF, MMP9, and FGF2 at significantly higher levels than N1 neutrophils. FGF2 stimulates endothelial cell production of MMPs and promotes the migration of endothelial cells and perivascular cells, suggesting the existence of specific subsets of neutrophils dedicated to promoting angiogenesis [66]. The 2017 study in “Nat Biomed Eng” demonstrated that N2 neutrophils in the host play an irreplaceable role in promoting vascular network formation between implants and the host. Depleting host neutrophils after implantation directly inhibits vascularization of the implant, while transferring N2 neutrophils to the host completely restores vascularization of the transplant [67]. Further research on neutrophil subtypes has recently identified a specific subgroup of neutrophils, CD49d^hi^ CXCR4^hi^ VEGFR1, that promotes angiogenesis in both humans and mice [51, 68]. VEGF‐A is capable of attracting a proangiogenic circulating group of neutrophils characterized by the presence of CD11b(+)/Gr‐1(+) markers. These neutrophils express high levels of CXCR4 and are responsible for delivering significant quantities of MMP9, an essential protein for revascularization and functional integration of transplanted islets [69]. N2 neutrophils polarized by HucMSC‐Exo promote the release of proangiogenic factors, specifically BV8 (a bone marrow cell‐derived proangiogenic factor), inducing angiogenesis and thus benefiting tissue repair [64]. As research progresses, more subtypes of neutrophils are being discovered, and their intricate functions in the body are continuously being explored. We believe that with further research on neutrophils in humans, their critical role in the field of regeneration will be better understood.

### 3.4 Excessive Activation of Neutrophils may Result in Vascular Injury

Semaphorin 4D (SEMA4D) regulates the activation of neutrophils in small blood vessels, and disruption of this regulation has been demonstrated to result in NET‐mediated vascular damage [70, 71]. Increased levels of soluble signaling protein 4D in serum are associated with disease activity in antineutrophil cytoplasmic antibody (ANCA)‐associated vasculitis (AAV) [70, 71]. Primary neutrophils express antigens specific to ANCA on their cell membranes, which subsequently bind with ANCA [72]. The FC‐γ receptor on neutrophils then interacts with the Fc region of ANCA, leading to excessive activation and subsequent abnormal production of cytokines, release of ROS and lysoenzymes. Ultimately, this results in the formation of NETs that contribute to the pathogenesis of AAV [72, 73].

## 4 Neutrophils Promote Tissue Injury Repair

**FIGURE 3:**
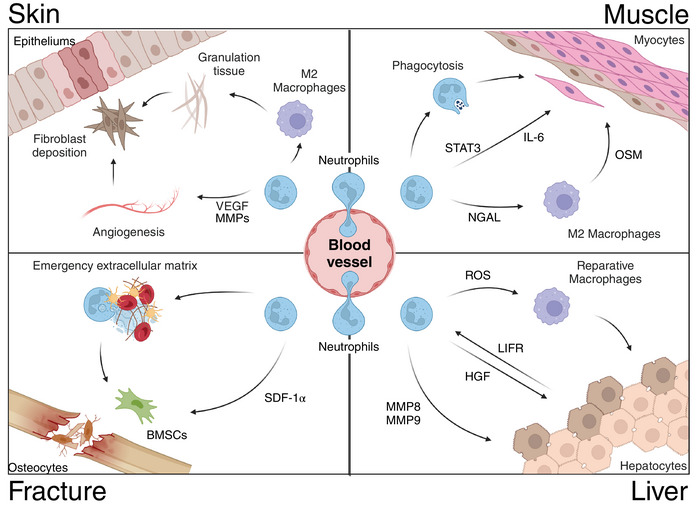
The role of neutrophils in different tissue repair. *Skin*: Neutrophils release VEGF or (MMPs) to stimulate angiogenesis and cell proliferation, while also secreting relevant cytokines that promote macrophage M2 polarization, thereby promoting granulation tissue formation and laying the foundation for fibroblast deposition of collagen to strengthen regenerative tissue. *Muscle*: Neutrophils can directly promote cardiomyocyte proliferation via interleukin‐6 (IL‐6) and the downstream signal transducer and activator of transcription 3 (STAT3) pathway. Additionally, they can mediate M2‐type differentiation of macrophages through neutrophil gelatinase‐associated lipocalin (NGAL), leading to the secretion of oncostatin M (OSM) and the establishment of a positive feedback loop, which in turn facilitates muscle growth and repair following injury. *Fracture*: A large number of neutrophils, linked to fibrinogen binding, are found in fracture hematoma. These neutrophils promote early healing by producing “an emergency extracellular matrix.” N2‐neutrophils release stromal cell‐derived factor‐1 (SDF‐1), recruiting bone marrow mesenchymal stem cells (BMSCs) for bone repair and regeneration. *Liver*: Neutrophils promote M2‐type macrophage differentiation via ROS, enhancing liver repair; and support repair by producing MMP8 and MMP9 which can resolve fibrosis. After liver injury, leukemia suppressor factor receptor (LIFR) mediates neutrophil recruitment and activation. Neutrophils then secrete hepatocyte growth factor (HGF) to accelerate liver regeneration. This figure was created using BioRender.com.

As research deepens, an interesting phenomenon has been discovered: despite the significant differences in structure and cell types among various organs, neutrophils have been found to play important roles in tissue repair in almost all organs (Figure 3) [6]. The presence of diverse subpopulations of neutrophils in tissue repair has been established [74]. For instance, in a murine model of myocardial ischemia‐reperfusion injury, neutrophils exhibiting high expression of Ym‐1 (Ym‐1^hi^Neu) demonstrate a reparative phenotype and their elevated expression is correlated with genes involved in the repair of injuries and angiogenesis [75]. Moreover, there exists a subset of CD14^+^ Ly6G^low^ neutrophils that express Arg1, mannose receptor C type 1, and IL‐4Ra while possessing neuroprotective and regenerative properties [76, 77]. It is currently believed that neutrophils mainly promote tissue repair by the following three strategies. First, neutrophils can eliminate necrotic cellular debris; second, Neutrophils release effectors that facilitate angiogenesis and regeneration processes; third, phagocytosis of apoptotic neutrophils leads to the secretion of anti‐inflammatory and reparative cytokines, characterized by the release of tissue‐repairing cytokines such as TGF‐β and IL‐10 [40].

### 4.1 Neutrophils’ Role in Skin Healing

The traditional view holds that since the skin is full of commensal and pathogenic bacteria, skin wounds are unlikely to be sterile. Therefore, it seems that the recruitment of neutrophils to skin injuries is unrelated to the injuries themselves. As the first cells to arrive at the site of injury, neutrophils combat the threat of infection through their classic functions of phagocytosis, degranulation, or the generation of ROS [7]. However, an increasing number of experiments have confirmed that neutrophils play a crucial role in promoting skin wound repair in addition to their classic antimicrobial functions. Clinical studies have found that patients with reduced neutrophil levels often suffer from delayed wound healing [4]. Similarly, research has found that in skin injury models of mice with defects in leukocyte surface G protein‐coupled formyl‐peptide receptors, the recruitment of neutrophils to the injury site is significantly reduced, leading to delayed wound healing, indicating that neutrophils also perform other functions in skin injury repair [78]. Furthermore, in a mouse model of neutrophil depletion, delayed skin wound healing was observed in elderly mice. However, this delayed healing phenomenon was reversed in elderly mice injected with G‐CSF (a glycoprotein that stimulates the production of neutrophils in the bone marrow and their release into the bloodstream), indicating the significant role of neutrophils in skin healing [79]. Additionally, as humans age, wound healing becomes slower, which is directly related to the decline in neutrophil function and the reduced efficiency of neutrophils in clearing infections [80]. Therefore, the important role of neutrophils in skin wound healing has been confirmed, and the quantity of neutrophils in the wound area is directly related to the speed of skin healing. A recent study published in *Nature* reports that neutrophils play a critical role in the reconstruction of the ECM in damaged skin, thereby enhancing both the mechanical properties and barrier function of the skin [81]. Hidalgo et al. [81] demonstrated through conjoint symbiosis experiments and bulk transcriptome analysis that neutrophils derived from barrier tissues such as the skin exhibit a distinct transcriptional signature associated with ECM remodeling.

After skin injury, the body immediately controls vasoconstriction through the sympathetic nervous system to control bleeding [82, 83]. Subsequently, a substantial recruitment of neutrophils occurs at the injury site, leading to the formation of a matrix‐rich ring composed of diverse ECM proteins surrounding the wound [81]. This structure effectively serves as a barrier to prevent the infiltration of harmful molecules and bacteria into the sterile tissue. Local neutrophils also promote thrombus formation by forming NETs [84]. Additionally, neutrophils release VEGF or MMPs to stimulate angiogenesis and cell proliferation, while also secreting relevant cytokines that promote macrophage M2 polarization, thereby promoting granulation tissue formation and laying the foundation for fibroblast deposition of collagen to strengthen regenerative tissue [7].

### 4.2 Neutrophils’ Role in Muscle Injury and Repair

Responding to tissue injury, muscles undergo tissue destruction and reconstruction, neutrophils play a pivotal role in both initiating muscle damage and facilitating the initial inflammatory response, ultimately enabling muscle repair. A research study revealed that, in the presence of TNF ‐ α, the CCL20–CCR6 axis is selectively activated to attract neutrophils expressing VEGF‐A with proangiogenic properties to areas affected by ischemic injury. This mechanism plays a crucial role in initiating revascularization of muscle tissue [85]. At present, research has indicated that neutrophils are the predominant cell infiltrating the muscle following an injury at a time immediately preceding the removal of necrotic tissue and activation of satellite cells [86]. It is conceivable that the facilitation of muscle repair by neutrophils could be attributed to their ability to eliminate tissue debris and stimulate satellite cells; furthermore, recent studies indicate that neutrophils may serve as a valuable source of IL‐6 and exert a direct influence on the proliferation of cardiomyocytes. This is accomplished through the activation of signal transducer and activator of transcription 3 (STAT3), an adapter protein acting downstream from the IL‐6 receptor. STAT3 plays a pivotal role in regulating satellite cell expansion and muscle repair [87].

A study found that neutrophils actively promote the repair of the heart muscle cell damage caused by a heart attack, and the underlying mechanism of promoting regeneration is that PMN mediates the differentiation of a distinct class of macrophages by producing a factor called neutrophil gelatinase‐associated lipocalin (NGAL), and macrophages play a key role in the repair process [88]. Additionally, a separate study demonstrated that neutrophils and macrophages release the cytokine oncostatin M (OSM), thereby establishing a positive feedback loop wherein OSM stimulates cardiomyocytes to generate regenerating islet‐derived protein 3 β (REG3β), which subsequently attracts additional macrophages to the injured heart [89]. More critically, one study found that the cocultivation of neutrophils with activated macrophages leads to a reduction in the secretion of proinflammatory molecules by macrophages by inhibiting the activation of NF‐κB, indicating that neutrophils polarize macrophages toward a reparative phenotype postmyocardial infarction [90]. In summary, neutrophils can also contribute to muscle growth and repair following injury.

### 4.3 Neutrophils’ Role in Fracture Healing

The process of fracture healing can be divided into four stages: hematoma formation, hematoma organization, callus formation, and callus remodeling [91]. It is known that a large number of neutrophils are present in the hematoma of a fracture. These cells arrive within minutes after the fracture, including mature neutrophils from circulation and immature neutrophils from the bone marrow [92]. Studies have found that local application of G‐CSF to increase neutrophil recruitment significantly accelerates fracture healing, while depletion of neutrophils significantly inhibits fracture healing [93]. Research has also discovered a significant amount of neutrophils associated with fibrinogen binding in the hematoma of patients with fractures. The authors propose a mechanism in which infiltrating neutrophils promote early fracture healing by producing “an emergency extracellular matrix” [94]. This suggests that neutrophils recruited by DAMPs, released after a fracture, are the first to arrive at the injured area. They can clear the microenvironment by phagocytosing pathogens and tissue or cellular debris. Subsequently, neutrophils interact with platelets to form NETs, promoting the formation of local hematoma. The arriving neutrophils then initiate remodeling of the local ECM and establish a vascular network to facilitate fracture healing. Recent studies on endogenous bone regeneration have also found that the neutrophil chemotactic factor IL‐8 can directly induce ectopic bone formation. This is mainly because an appropriate concentration of IL‐8 can recruit a large number of N2‐polarized neutrophils to the defect area and recruit BMSCs) through stromal cell‐derived factor‐1 (SDF‐1) released by N2 neutrophils to participate in bone tissue repair and regeneration [8]. Numerous studies have shown that neutrophils participate in bone tissue repair by improving local inflammation, remodeling the ECM (hematoma) through MMP9, recruiting stem cells through the secretion of stem cell recruiting factors, and guiding vascular network construction [94]. However, further research is needed to understand the specific mechanisms of neutrophil involvement in each stage. Bone is one of the few organs in the body that can undergo extensive damage repair and achieve complete restoration without fibrosis [95]. Neutrophils themselves are produced in the bone marrow, stored within the bone marrow cavity, and are abundant in bone [96]. Therefore, the intriguing topic of whether the strong regenerative capacity of bone is related to the abundance of neutrophils in the bone marrow cavity, and whether poor bone healing and related diseases are associated with changes in the quantity and quality of neutrophils, deserves further consideration.

### 4.4 Neutrophils’ Role in Liver Tissue Repair

The liver, renowned for its remarkable regenerative capacity, is frequently employed in tissue repair experiments. In the model of liver thermal injury, hepatic stellate cells are observed as the initial responders to the site of injury, facilitating hepatic repair by dismantling the barrier surrounding the injured area and guiding neovascularization into this region [44]. Depletion of neutrophils leads to an accumulation of cellular debris and delayed vascular reconstruction, ultimately impeding liver healing [97]. Similarly, a recent investigation has elucidated the dual role played by neutrophils in acute liver injury models: while they initially contribute to damage through removal of impaired cells, their absence during the reparative phase exacerbates liver impairment [98]. Furthermore, it has been discovered that neutrophils inhibit CCl4‐induced fibrosis development in chronic liver injuries and promote hepatic repair via production of metalloproteinases (MMP8, MMP9) that facilitate fibrolysis [99]. It has also been shown that neutrophils trigger macrophage skewing toward a reparative phenotype for optimal liver repair and the process is mediated by ROS [100]. The latest study also revealed the mutual response of liver cells and neutrophils after liver damage. Leukemia inhibitory factor receptor (LIFR) forms bidirectional hepatocyt–neutrophil cross‐talk via LIFR–STAT3–CXCL1–CXCR2 axis and a LIFR–STAT3–cholesterol–ERRα–hepatocyte growth factor (HGF) axis to repair and regenerate liver [101]. Upon physical or chemical injury to the liver, LIFR facilitates STAT3‐dependent secretion of CXCL1 and cholesterol, thereby recruiting and activating neutrophils. Subsequently, these neutrophils secrete HGF to expedite hepatic repair and regeneration [101]. Evidently, neutrophils play a pivotal role in safeguarding and promoting recovery from liver injuries.

## 5 Fate of Neutrophils and the subsequent Impact on Tissue Repair

**FIGURE 4:**
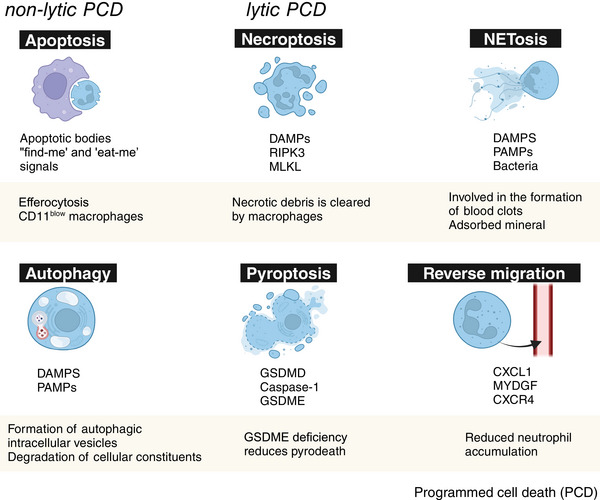
Fate of neutrophils and the subsequent impact on tissue repair. The modes of neutrophil death include nonlytic PCD (apoptosis and autophagy), lytic PCD (necroptosis and pyroptosis), NETosis, and reverse migration. Neutrophil apoptosis signals “find me” and “eat me,” leading to macrophage clearance via efferocytosis and promoting a CD11^blow^ proresolution macrophage subset. Autophagy is triggered by pattern‐recognition receptors, forming intracellular vesicles and degrading cellular components. Necroptosis depends on receptor‐interacting protein kinase‐3 (RIPK3) and mixed lineage kinase domain‐like protein (MLKL), releasing damage‐associated molecular patterns (DAMPs) that exacerbate tissue injury, with products phagocytosed by macrophages. Pyroptosis requires Gasdermin D (GSDMD) and caspase‐1, while Gasdermin E (GSDME) loss specifically inhibits pyroptosis of neutrophils. DAMPs, pathogen‐associated molecular patterns (PAMPs), and bacterial can induce NETosis, where neutrophil extracellular traps (NETs) promote blood clotting and early tissue repair. CXCL1, myeloid‐derived growth factor (MYDGF), and CXCR4 drive reverse migration, reducing N1‐neutrophils, resolving inflammation, and supporting tissue regeneration. This figure was created using BioRender.com.

Neutrophils are cleared through diverse mechanisms, which further influence immune responses, tissue repair, and regeneration [7]. For example, they can undergo apoptosis or necrosis and then be engulfed by macrophages, or they can migrate back to the bloodstream or be expelled into the external environment [102]. The clearance of neutrophils is crucial for the resolution of inflammation in the injury site, directly impacting the outcome of tissue repair (Figure 4) [40].

### 5.1 Apoptosis and Efferocytosis

Apoptosis is a gene‐controlled programmed cell death process that cells undergo to maintain internal environmental stability. Neutrophils undergoing apoptosis expose phosphatidylserine on their surface, which is recognized by macrophages, inducing a series of proresolution cascade reactions, including the release of repair cell factors such as TGF‐β, IL‐10, and VEGF, leading to the reprogramming of macrophages into an anti‐inflammatory M2 phenotype [40, 103]. In addition, macrophages release proteases and lipoxins, further enhancing their phagocytic activity toward apoptotic neutrophils [104]. Moreover, α‐defensins released by apoptotic neutrophils increase the phagocytic ability of macrophages and suppress their release of inflammatory mediators [105]. Recent studies have also found that apoptotic bodies, as the final product of cell apoptosis, play a very important role in tissue repair and inflammation control. The apoptotic bodies of neutrophils in the injured area play a positive role in the resolution of local inflammation, as neutrophils undergoing apoptosis can slow down local inflammation. Meanwhile, the released apoptotic bodies can be engulfed by macrophages as signals for injury repair, promoting M2 polarization of macrophages to alleviate local inflammation and facilitate tissue repair [100]. In summary, apoptosis is an important, noninflammatory form of cell death and one of the main mechanisms by which neutrophils in the damaged area control the resolution of inflammation, which is beneficial for promoting tissue repair. Research has shown that when neutrophils undergo apoptosis, they release lysophosphatidylserine, which is recognized by type 3 innate lymphoid cells (ILC3s) through GPR34 activation. This leads to the activation of ILC3s and initiation of epithelial repair [106]. In vivo experiments using mice with a conditional knockout of GPR34 in ILC3s and neutrophil‐depleted wild‐type mice have demonstrated impaired healing due to decreased IL‐22 expression [106]. In addition, phagocytosis induces neutrophil apoptosis, which expresses “find‐me” and “eat‐me” signals and is removed by macrophages via efferocytosis, which would further lead to polarization and reprogramming of macrophages toward a promoting repair phenotype [107].

A potential positive impact for neutrophils in tissue repair has been identified, that can be directly mediated through the clearance of cellular debris and the release of growth factors, anti‐inflammatory molecules, and proangiogenic factors, or through the process of macrophage‐mediated engulfment of dying neutrophils (efferocytosis). Instead of necrosis, which results in the release of intracellular components and triggers an immediate inflammatory reaction, neutrophil apoptosis involves the exposure of phosphatidylserine on the outer surface of the cell. This exposure serves as a signal for macrophage efferocytosis [108].

The elimination of apoptotic neutrophils (as well as other cellular entities) through efferocytosis by macrophages plays a crucial role in concluding the inflammatory response. Furthermore, in the process of efferocytosis, phagocytosis of apoptotic neutrophils by macrophages stimulates the production of anti‐inflammatory and proresolving mediators, including IL‐10, TGF‐β1, VEGF, and specialized proresolving mediators [109, 110], contributing to inflammation resolution and tissue repair. In addition, Apoptotic cells secrete “find‐me” signals, such as ATP and UTP, to attract monocytes/macrophages toward their vicinity [111], along with “eat‐me” signals like phosphatidylserine, that facilitate recognition and engulfment [112]. And lactoferrin, released by apoptotic cells, acts as a “keep‐out” signal, it inhibits granulocyte trafficking while allowing for monocyte recruitment, thus restricting the extent of inflammation [113]. Efferocytosis induces a phenotypic switch in macrophages from the M1 proinflammatory state to the M2 anti‐inflammatory state, thereby initiating the secretion of reparative cytokines such as TGF‐β and promoting inflammation resolution [112], and subsequently to a CD11b^low^ proresolution subset [114]. CD11^blow^ macrophages display limited phagocytic activity and secrete IFN‐β, which mediates reciprocal communication between neutrophils and macrophages to promote neutrophil apoptosis and efferocytosis, thereby expediting resolution [115].

### 5.2 Autophagy

Autophagy, which is conserved throughout evolution, promotes cell homeostasis by degrading and recycling intracellular components in response to stress or nutrient deprivation, and autophagy is characterized by the formation of autophagic intracellular vesicles and the degradation of cellular constituents [116]. Many of the PRRs, including TLRs and NLRs, induce autophagy in macrophages [117]. Similarly, in the presence of TLR ligands, neutrophils exhibit autophagy. Autophagy can be triggered in neutrophils either through phagocytosis or independently of it [118].

Survival in the neutrophil is facilitated through autophagy. It has been noted that the presence of autophagy‐related morphological features in neutrophils during septic shock, rheumatoid arthritis, and different skin conditions implies that the activation of autophagy in PMN is a widespread occurrence in their inflammatory response [118]. In addition, it has also been demonstrated that the suppression of autophagy decreased NETosis, preventing chromatin decondensation and resulting in cell demise characterized by signs of apoptosis [119]. The fact that autophagy can be triggered in neutrophils by LPS or phagocytosis of opsonized particles, regardless of NET formation, suggests the existence of an independent mechanism for combating pathogens [120]. Hence, autophagy plays a pivotal role as a crucial regulatory mechanism in neutrophil function, exerting direct influence on cellular differentiation, phagocytosis, cytokine production, degranulation, IL‐1β synthesis, bacterial eradication, and cell death [116]. Furthermore, autophagic cell death has the potential to regulate the recruitment of neutrophils to inflammatory sites, thereby mitigating excessive tissue damage or the progression of persistent inflammation [102].

### 5.3 Necrosis and Necroptosis

Necrosis is an intense, uncontrolled form of cell death, characterized by cell swelling, membrane rupture, mitochondrial depolarization, oxidative burst, and release of cell contents. Neutrophil necrosis releases a large number of DAMPs, activating TLRs on immune cells, thereby inducing the activation of NF‐κB in macrophages and fibroblasts, stimulating the production of proinflammatory factors such as IL‐1, neutrophil chemotactic factors, and exacerbating the inflammatory response and local tissue damage. The products of neutrophil necrosis itself can also aggravate tissue damage [121, 122]. To deal with these necrotic products and alleviate their damage to the tissues, the body processes them in different ways. One way is through the clearance of the remnants of necrotic cells and the absorption of released products by phagocytic cells (such as macrophages). Another way is to control the inflammatory factor response and promote tissue repair through inflammation regulation mechanisms [123].

Necroptosis is a form of programmed cell death, characterized by its regulated nature and dependence on receptor‐interacting protein kinase‐3 (RIPK3) and mixed lineage kinase domain‐like protein [124]. In addition, although necroptosis is a form of programmed cell death, it lacks the precise morphological changes characteristic of apoptosis and, importantly, does not involve DNA fragmentation. Moreover, various mechanisms have been identified to trigger this process in neutrophils. Neutrophils have the potential to undergo necroptosis simultaneously with or without NET extrusion. Research has shown a correlation between necroptosis and the release of NETs in the presence of monosodium urate. This particular stimulus is associated with the generation of ROS through the initiation and functioning of the NADPH oxidase complex. However, the adhesion molecules are unable to facilitate necroptosis in neutrophils of patients with chronic granulomatous disease due to their inability to produce intracellular ROS [124, 125]. More critically, it was noted that the suppression of RIPK3, as well as the effects of other necroptosis inhibitors, decreased the formation of NETs induced by monosodium urate crystals, thus confirming the relation of necroptosis with NETosis, at least after this stimulus [126].

### 5.4 Pyroptosis

Pyroptosis can be described as a type of programmed cellular demise triggered by the presence of intracellular pathogens, and this pathway of cell death is dependent on Gasdermin D and caspase‐1 [127, 128]. Moreover, this specific caspase does not play a role in initiating programmed cell death; instead, its primary function lies in facilitating the processing of precursor molecules for the inflammatory cytokines IL‐1β and IL‐18. This process takes place within a complex known as the inflammasome, which is crucial for the execution of pyroptosis [126]. Although neutrophils are especially resistant to pyroptosis, in cases of acute lung infection caused by *P. aeruginosa*, it has been observed that pyroptosis may occur as evidenced by the activation of an alternative inflammasome pathway through P2X7 receptors in the absence of functional NADPH oxidase Nox2. It is hypothesized that this compensatory mechanism could be triggered to counterbalance the deficiency in antimicrobial pathways [129]. Since neutrophils play an important role in sepsis, it has been suggested that the regulation of neutrophil pyroptosis may positively impact sepsis treatment [130]. Recently, Luo et al. [131] have identified Gasdermin E (GSDME) as a pivotal regulator of neutrophil lytic pyroptotic death. They discovered that the absence of GSDME specifically inhibits pyroptosis and redirects neutrophil death toward apoptosis, thereby attenuating the inflammatory response triggered by enhanced macrophage exocytosis against apoptotic neutrophils. In models of acidic aspiration lung injury and bacterial pneumonia, GSDME‐deficient neutrophils exhibited diminished lung inflammation and injury. This not only provides novel insights into the functionality and regulatory mechanisms of neutrophils but also presents innovative therapeutic strategies for inflammatory diseases.

### 5.5 Neutrophil Extracellular Traps

NETs are a special form of cell death exhibited by neutrophils. NETs are released by neutrophils and are composed of chromatin scaffolds condensed with cytoplasmic proteins, granule proteins, and other components. They form a sticky extracellular mesh‐like structure. Initially discovered in the context of host defense, NETs are used to capture and kill trapped pathogens [84, 132]. The neutrophil death mediated by NETs releases proinflammatory and chemotactic factors, leading to the recruitment of activated neutrophils and monocytes to the site of tissue damage and exacerbating local inflammation. Accumulation of stimulated immune cells, in combination with ongoing production of NETs, can form blood clots, and in severe cases, may lead to disseminated intravascular coagulation [133]. Studies have shown that excessive NETs can contribute to a dysregulated inflammatory environment, impair functional tissue healing, and even lead to local thrombosis and tissue fibrosis, thereby exacerbating local tissue damage [134]. However, for early tissue damage, NETs can promote blood clot formation through different mechanisms. Activated NETs can concentrate the effector proteins involved in blood clot formation while providing a scaffold for platelet and red blood cell adhesion. Platelets are activated by DNA‐bound histones and aggregate to form blood clots, providing a material basis (scaffold structure) for early tissue repair [135, 136, 137]. Studies have also found that NETs released by neutrophils in the injury area can adsorb and capture growth factors, ECM proteins, and other cytokines that promote bone healing, providing an appropriate microenvironment for tissue repair [132]. Recent research has also found that NETs themselves can act as a scaffold structure, adsorbing and aggregating the minerals and organic substances required for mineralization, promoting the formation of bone and tooth tissue [138]. With further research on NETs, more functions in regeneration will be discovered.

### 5.6 Reverse Migration

Traditional views hold that neutrophils have a lifespan of only 1.5–8 h, but recent studies have found that the average circulating lifespan of neutrophils can reach 5.4 days [139]. Therefore, people have begun to widely recognize that neutrophils do not always die at the site of injury, but can leave the site of injury and return to the vascular system. Studies have used in vivo imaging techniques to directly observe that in aseptic liver injury mouse models, neutrophils re‐enter the vascular system, pass through the lungs, then upregulate the expression of the chemokine SDF‐1 (also known as CXCL12) and its specific receptor CXCR4 (C‐X‐C motif chemokine receptor 4) before returning to the bone marrow, where they are ultimately cleared [140]. Additionally, experiments in rat models of renal glomerular capillary injury have shown that more than 70% of neutrophils entering the injured area of the renal glomerular capillaries can return to the main circulation without undergoing apoptosis at the site of injury [141]. These results all demonstrate that the reverse migration of neutrophils is common and can help reduce local inflammatory reactions by controlling the number of neutrophils in the injured area.

C‐X‐C chemokine ligand 1 (CXCL1) is considered to be one of the key cell factors driving the reverse migration of neutrophils. Studies have shown that the concentration of CXCL1 in the blood increases during the neutrophil reverse migration stage, and this concentration significantly promotes the reverse migration of neutrophils [142, 143]. In addition, the secretion of myeloid‐derived growth factor (MYDGF) by macrophages is also considered a key cell factor guiding the reverse migration of neutrophils [144, 145]. Current research suggests that during the reverse migration process, the majority of neutrophils return to the bone marrow through the main circulation, primarily driven by reverse migration factors such as CXCL1 or MYDGF. Neutrophils first return to the vascular system, where neutrophils expressing high levels of CXCR4 then, under the regulation of CXCL12/CXCR4 signaling, ultimately return to the bone marrow [140]. Of course, a small portion of neutrophils will enter other tissues through circulation, such as lung tissue. Compared with neutrophils from nondamaged areas, neutrophils from the injured area will firmly adhere to pulmonary endothelial cells, stay for a longer time, and cause pulmonary inflammatory responses. In severe cases, these neutrophils may be involved in the development of acute respiratory distress syndrome. However, the specific mechanism is not yet fully understood [140]. We believe that reverse migration may be one of the most efficient and physiologically relevant ways to reduce the number of neutrophils in the injured area. The reverse migration of neutrophils from the damaged area can rapidly reduce the number of N1‐type neutrophils, promote the resolution of local inflammation, and create a favorable microenvironment for tissue regeneration and repair [146, 147]. The inhibition of reverse migration may be associated with poor outcomes of tissue repair due to excessive activation of local inflammation.

## 6 Conclusion and Outlook

In conclusion, neutrophils play crucial roles in the early inflammatory response, establishment of the regenerative microenvironment, tissue repair and regeneration, and resolution of local inflammation in the injured area, and the process of neutrophil‐related tissue repair involves the participation of multiple cytokines at various stages (Table 1). During the early inflammatory phase following injury, neutrophils are the first to infiltrate the damaged area, laying the foundation for tissue repair by eliminating invading microorganisms and clearing tissue debris. Subsequently, through the secretion of MMPs, they remodel the ECM in the injured area and secrete various cytokines to create a reparative and regenerative microenvironment, facilitating the subsequent recruitment of new blood vessels and stem cells. Finally, some neutrophils leave the injured area through reverse migration, while others contribute to the recovery of local inflammation levels by promoting M2 differentiation of macrophages through some forms of cell death such as apoptosis.

**TABLE 1:** Cytokines involved in neutrophil‐promoting tissue repair.

Abbreviation	Terms	Paraphrase
DPEP1	Dipeptidase ‐ 1	A 55‐kDa glycosyl‐phosphatidyl‐inositol (GPI) anchored membrane protein [153]. DPEP1 functions as an adhesion receptor for neutrophils within the liver and pulmonary vascular system, following stimulation by bacterial endotoxin lipopolysaccharide (LPS) [15].
Myd88	Myeloid differentiation factor 88	MyD88 is a Toll/IL‐1 receptor (TIR) that resides in the cytoplasm and binds toTLR4 upon LPS binding [154].
NLRP3	NOD‐like receptor thermal protein domain associated protein 3	NLRP3, also referred to as cryopyrin, is a member of the nucleotide‐binding domain and leucine‐rich repeat (NLR) protein family. It has been identified in immune cells such as neutrophils, monocytes, and dendritic cells within the bone marrow [155].
CGRP	Calcitonin gene‐related peptide	A 37 amino acid peptide belonging to a superfamily of peptides including CT, α‐CGRP, β‐CGRP, amylin (AMY), adrenomedullin (ADM), and the recently discovered intermedin (IMD) and calcitonin receptor‐stimulating peptide [156].
Mrgpra1	Mas‐related of G‐protein‐coupled receptor a1	Mrgpra1 is expressed in sensory neurons innervating the skin and regulates the itch and pain response [157, 158]. It has been shown to bind to a variety of ligands, including RF amide neuropeptides (NP) such as NPFF, Substance P (SP), somatostatin, and bilirubin [157, 159].
NPFF	Neuropeptide FF	Phe–Leu–Phe–Gln–Pro–Gln–Arg–Phe–NH2. Neuropeptide FF is an 8‐amino acid peptide found in laminae I and II of the dorsal horn [160].
BV8		BV8 also known as prokineticin 2 (Prok2), is a bone marrow cell‐derived proangiogenic factor [161].
SEMA4D	Semaphorin 4D	A transmembrane protein that has been shown to have activities on vascular endothelial cells [162].
NGAL	Neutrophil gelatinase‐associated lipocalin	NGAL is a lipid carrier protein that has the function of transporting hydrophobic small molecules. It can bind to MMP‐9 to regulate the function of MMP‐9. It is involved in the clearance of inflammatory mediators and immune regulation [163].
OSM	Oncostatin M	OSM is a member of the IL‐6 cytokine family and can be synthesized by monocytes, macrophages, neutrophils and activated T cells [89]. OSM is also a central mediator of cardiomyocyte dedifferentiation and protects the heart after myocardial infarction [164].
LIFR	Leukemia inhibitory factor receptor	LIFR is abundant in the liver and it has an essential role in development [101].
GSDME	Gasdermin E	GSDME is a member of the gasdermin family that encodes a homologous protein [131]. Its active form, triggered by caspase‐3, facilitates cell pyroptosis, release of proinflammatory substances, and generation of a proinflammatory microenvironment [165].

Over the years, the majority of therapeutic targets have focused on attenuating the proinflammatory effects of neutrophils and modulating the inflammatory microenvironment to facilitate tissue endogenous repair and regeneration (Table 2). Currently, there is a growing body of research focused on enhancing the ability of neutrophils to reduce inflammation during the initial stages. For example, Segal et al. [76, 77] found that IL‐4/G‐CSF polarized mouse and human bone marrow neutrophils display increased activation markers and produce various growth factors, enabling them to support the growth of nerve fibers. On the other hand, Zigmond et al. [148, 149] observed that damaged nerve cells release CXCL1 and CXCL2 to attract neutrophils and promote their interaction with phagocytes for cell fragmentation. These discoveries have significant implications for potential therapeutic interventions aimed at repairing nerve cells affected by neurodegenerative diseases such as Alzheimer's and Parkinson's. It can be seen that the potential to harness the early anti‐inflammatory role of neutrophils for promoting tissue regeneration is evident.

**TABLE 2:** Therapeutic targets for neutrophils that modulate the inflammatory microenvironment to orchestrate tissue repair.

Mechanism		Target	Indication	References
Recruitment	Chemokine receptor inhibitors	CXCR1/2	Periodontitis	[166]
	Cytokine antagonists	G‐CSF	Rheumatoid arthritis	[167, 168]
Inflammatory mediators	Cytokine antagonists	TNF‐α, IL‐1β	Rheumatoid arthritis	[153]
	Proresolving mediators	Resolvins	Alzheimer's disease	[169]
Inhibit cytotoxic effects	Degranulation inhibitor	Neurokinin receptor	Rheumatoid arthritis	[170, 171]
	Respiratory burst inhibitors	NADPH oxidase	Ischemia reperfusion injury	[172]
	NETs inhibitors	PAD4	Colitis	[173]
Survival and migration	Apoptosis inducers	Roscovitine, INF‐β	Myocardial infarction	[174, 175]
	Prosurvival signaling inhibitors	PI3K	Rheumatoid arthritis	[176]

*Abbreviations*: CXCR1/2: CXC‐chemokine receptor 1/2; G‐CSF: granulocyte colony‐stimulating factor; IL‐1β: interleukin‐1β; INF‐β: Interferon‐β; PAD4: protein arginine deiminase 4; PI3K: phosphatidylinositol 3‐kinase; TNF‐α: tumor necrosis factor‐α.

As previously mentioned, neutrophils demonstrate significant phenotypic and functional adaptability, generating interest in their reprogramming for personalized medicine, especially via mechanisms linked to circadian rhythms [150]. The circadian control of innate neutrophil activation, together with the observation that neutrophils alternate between high and low inflammatory states, indicates a potential therapeutic approach [150, 151]. A myeloid‐specific, heritable deletion of the core circadian gene Bmal1 disrupts the balanced circadian movement of neutrophils, a process further influenced by a unique set of receptors in endothelial cells and neutrophils [152]. Inhibiting these receptors reduces inflammation in response to systemic LPS challenges in mice. These results highlight the possibility of utilizing the time‐based physiology involved in neutrophil reprogramming and migration for therapeutic purposes.

The past decade has been a golden age for neutrophil biology research. Neutrophils are no longer seen as just suicidal cells that trigger acute inflammation and tissue damage; an increasing number of studies have found that they play a crucial role in tissue repair and regeneration. We believe that with further in‐depth research into the biological mechanisms of neutrophils, precise control over neutrophil function will emerge as a novel strategy and approach for organ repair and tissue engineering in the future.

## Author Contributions

**Luying Yang** and **Fan Shi**: writing – original draft; **Feng Cao**: writing – review and editing; **Le Wang, Jianzhen She, Boling He, Xiaoying Xu**: formal analysis, resources; **Liang Kong**: project administration, supervision; **Bolei Cai**: conceptualization; writing – review and editing, funding acquisition. All authors have read and approved the final manuscript.

## Ethics Statement

The authors have nothing to report.

## Conflicts of Interest

The authors declare no conflicts of interest.

## Data Availability

No data were used for the research described in the article.
